# Supplementary material for: Streptomyces exploration is triggered by fungal interactions and volatile signals
Source: eLife. 2017 Jan 3;6:e21738. doi: 10.7554/eLife.21738 (PMC5207766; doi:10.7554/eLife.21738)
Supplement: Supplementary file 1. — (a) VOCs identified using GC×GC-TOFMS. (b) Effects of media composition on S. venezuelae exploration when grown in the absence of yeast. (c) Oligonucleotides used in this study. DOI: http://dx.doi.org/10.7554/eLife.21738.021 [file elife-21738-supp1.docx]

**SUPPLEMENTARY FILE 1**

**Supplementary File 1a. VOCs identified using GC×GC-TOFMS.**

| **Compound** | **G-**  **TIC average** | **G+**  **TIC average** | **% of**  **G- samples** | **% of**  **G+ samples** |
| --- | --- | --- | --- | --- |
| 1,5-Heptadiene, 2,5-dimethyl-3-methylene- | 157486 | 909 | 69.00 | 83.33 |
| 2,5-Cyclohexadien-1-one, 4-ethyl-3,4-dimethyl- | 58829 | 0 | 53.85 | 0 |
| 2-Acetylthiazole | 27658 | 0 | 100.00 | 0 |
| 2-Methylisoborneol | 3963625 | 76398 | 100.00 | 100 |
| 3-Caren-10-al | 111806 | 354 | 53.85 | 33.33 |
| 4-Hydroxy-3-hexanone | 10995 | 0 | 53.85 | 0 |
| 5-Hepten-2-one, 6-methyl- | 13963 | 0 | 100.00 | 0 |
| Acetamide, N-(2-methylpropyl)- | 59056 | 0 | 61.54 | 0 |
| Acetonitrile, (dimethylamino)- | 4501 | 0 | 76.92 | 0 |
| Aniline, N-methyl- | 1084056 | 43298 | 100.00 | 100 |
| Butanoic acid, 3-methyl- | 64178 | 1273 | 69.23 | 16.67 |
| Dimethyl trisulfide | 4289376 | 43303 | 100.00 | 100 |
| Disulfide, dimethyl | 15430045 | 460968 | 100.00 | 100 |
| Disulfide, methyl (methylthio)methyl | 5610 | 0 | 53.85 | 0 |
| Furan, 2-methyl- | 33530 | 1608 | 100.00 | 100 |
| Hexanenitrile | 5956 | 0 | 84.62 | 0 |
| N-(3-Methylbutyl)acetamide | 37841 | 0 | 92.31 | 0 |
| Propane, 1-bromo-2-methyl- | 4981 | 218 | 53.85 | 16.67 |
| Tetrasulfide, dimethyl | 24737 | 0 | 76.92 | 0 |
| Thiocyanic acid, methyl ester | 43203 | 0 | 100.00 | 0 |
| Trimethylamine | 453549 | 0 | 100.00 | 0 |

TIC = Total Ion Chromatogram (GC peak area, = measure of abundance)

**Supplementary File 1b. Effects of media composition on *S. venezuelae* exploration when grown in the absence of yeast.**

|  | Glucose represses exploratory behaviour | | | | | A peptide source is required to induce exploratory behaviour | | | | | | Various peptide sources induce exploratory behaviour | |
| --- | --- | --- | --- | --- | --- | --- | --- | --- | --- | --- | --- | --- | --- |
| Maltose |  |  |  |  |  |  | ✔ | ✔ | ✔ |  |  |  |  |
| Yeast extract |  | ✔ | ✔ |  | ✔ | ✔ | ✔ | ✔ | ✔ | ✔ |  | ✔ | ✔ |
| Malt extract |  |  |  |  |  |  |  |  |  |  |  |  |  |
| Amino acid source* |  | ✔(p) |  | ✔(p) | ✔(p) | ✔(p) | ✔(p) |  | ✔(p) |  | ✔(p) | ✔(c) | ✔(t) |
| Glucose | ✔ | ✔ | ✔ | ✔ | ✔ |  |  |  |  |  |  |  |  |
| pH | 6 | 5 | 5 | 5 | 5 | 9 | 9.5 | 6.5 | 8.5 | 8 | 8 | 9.5 | 9.5 |
| Exploratory behaviour |  |  |  |  |  | ✔ | ✔ |  | ✔ |  |  | ✔ | ✔ |

*: p = peptone, c = casaminoacids, t = tryptone

**Supplementary File 1c. Oligonucleotides used in this study**

| **Name** | **Sequence (5′ to 3′)** | **Use** |
| --- | --- | --- |
| Sven3715 Up | TCAAGATCATGACCTGGTGC | Confirmation of ∆*cydCD* mutation |
| Sven3715 Down | CAGGAGCTGGGGCACTCGG | Confirmation of ∆*cydCD* mutation |
| Sven3715 in | CTTCTGGAAGGACCCCACC | Confirmation of ∆*cydCD* mutation |
| Sven3715 Fwd | CGCCGAGACCCACTAGCCGGTCCTGTCCAGGGAGCAATGATTCCGGGGATCCGTCGACC | Creation of ∆*cydCD* strain; confirmation of ∆*cydCD* mutation |
| Sven3715 Rev | GACGCGGCGGCGGTCATGGCTTGAGCCTAGTAAGTCCTATGTAGGCTGGAGCTGCTTC | Creation of ∆*cydCD* strain; confirmation of ∆*cydCD* mutation |
| rpoBPF | GAGCGCATGACCACCCAGGACGTCGAGGC | Amplification of *rpoB* from WAC strains and *S. venezuelae* |
| rpoBPR | CCTCGTAGTTGTGACCCTCCCACGGCATGA | Amplification of *rpoB* from WAC strains and *S. venezuelae* |
| rpoBF1 | TTCATGGACCAGAACAACC | Sequencing of *rpoB* from WAC strains and *S. venezuelae* |
| rpoBR1 | CGTAGTTGTGACCCTCCC | Sequencing of *rpoB* from WAC strains and *S. venezuelae* |
